# The Role of Quality Control in Targeted Next-generation Sequencing Library Preparation

**DOI:** 10.1016/j.gpb.2016.04.007

**Published:** 2016-07-28

**Authors:** Rouven Nietsch, Jan Haas, Alan Lai, Daniel Oehler, Stefan Mester, Karen S. Frese, Farbod Sedaghat-Hamedani, Elham Kayvanpour, Andreas Keller, Benjamin Meder

**Affiliations:** 1 Institute for Cardiomyopathies, Department of Internal Medicine III, University of Heidelberg, 69120 Heidelberg, Germany; 2 German Centre for Cardiovascular Research (DZHK), Heidelberg/Mannheim, Germany; 3 Chair for Clinical Bioinformatics, Medical Faculty, Saarland University, 66123 Saarbrücken, Germany

**Keywords:** Next-generation sequencing, Quality control, Library preparation, Target enrichment, Sequence variants

## Abstract

**Next-generation sequencing** (NGS) is getting routinely used in the diagnosis of hereditary diseases, such as human cardiomyopathies. Hence, it is of utter importance to secure high quality sequencing data, enabling the identification of disease-relevant mutations or the conclusion of negative test results. During the process of sample preparation, each protocol for **target enrichment library preparation** has its own requirements for **quality control** (QC); however, there is little evidence on the actual impact of these guidelines on resulting data quality. In this study, we analyzed the impact of QC during the diverse **library preparation** steps of Agilent SureSelect XT **target enrichment** and Illumina sequencing. We quantified the parameters for a cohort of around 600 samples, which include starting amount of DNA, amount of sheared DNA, smallest and largest fragment size of the starting DNA; amount of DNA after the pre-PCR, and smallest and largest fragment size of the resulting DNA; as well as the amount of the final library, the corresponding smallest and largest fragment size, and the number of detected variants. Intriguingly, there is a high tolerance for variations in all QC steps, meaning that within the boundaries proposed in the current study, a considerable variance at each step of QC can be well tolerated without compromising NGS quality.

## Introduction

Before the advent of next-generation sequencing (NGS), genetic testing was realized by Sanger sequencing [1], which meant analyzing a gene exon-wise or amplicon-wise in a relatively elaborate, time-consuming and costly way. This substantially limited the number of genes that could be examined in parallel. In 2005, the first commercial NGS systems were introduced, yielding up to 20 megabase (mb) output per run [2]. Genetic studies have gained enormously from NGS over the past years. There is no doubt that NGS has matured to a technique that is highly reliable if performed by following certain rules [3]. Today, it is to replace Sanger sequencing not only in research, but also in clinical applications. One major step in this path is the first marketing authorization for an NGS instrument (Illumina’s MiSeqDx) by the Food and Drug Administration of the United States (US FDA) [4]. Besides such optimism, less certainty exists on the required standards for ensuring sequencing quality. It is also debated whether precise bench-work or careful data analysis is more important. For gene panel or target enrichment, a number of distinct protocols based on, *e.g.*, PCR, hybridization, or selective circularization, have been developed [5]. For each of these methods, stringent quality control (QC) steps were introduced to ensure a consistent data quality of the resulting NGS process. On the other hand, QC is expensive and requires significant hands-on time. Moreover, it is virtually unknown how QC could affect the sequencing process in case of abnormal results obtained.

With respect to the influence of data analysis on sequence quality, numerous studies and recommendations provide a guideline toward reproducible and comparable NGS results [6]. This so-called post-sequencing QC typically starts with raw-data processing covering measures of base quality, nucleotide distribution, GC content distribution, and read duplication rate. Then post-alignment QC is mostly based on the BAM-files, which provides QC parameters like the number of mappable reads, mapping quality, depth of coverage, and the number of reads mapped to the target region. Finally, on the variant level, data quality can be analyzed by the transition/transversion (Ti/Tv) ratio, heterozygosity rate, or occurrence in variant databases [3].

In this study, we investigated, using a large-scale dataset from nearly 600 patients, the impact of the many different QC phases during library preparation on the resulting sequencing data, and provided a recommendation on library quality requirement.

## Results and discussion

### Impact of library preparation on NGS quality

While it is broadly appreciated that post-processing of sequencing data is inevitable, less certainty exists on the influence of wet-lab steps during library preparation on the final quality of variant calls. Hence, we collected data from stringent QC during a larger-scale diagnostic target-enrichment study, which has underlined the high analytical quality and feasibility of NGS in a clinical genetic diagnostic setting [3].

**Figure 1:**
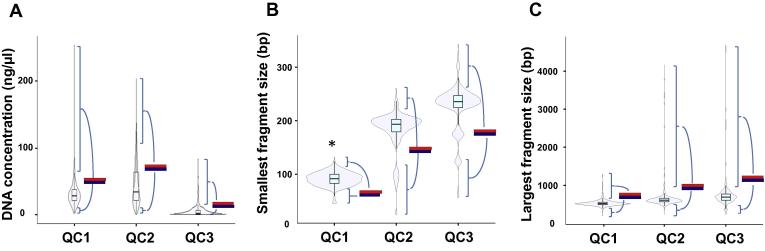
**QC-parameter distribution** The violin plots depict the distribution of parameters concentration (ng/μl; **A**), smallest fragment length (bp; **B**), and largest fragment length (bp; **C**), at the three steps of enrichment and library preparation QC1, QC2, and QC3. The number of total variant calls for the top 10% of each parameter is plotted as the red horizontal bar or as blue horizontal bar for the bottom 10%, and then compared using *t*-test. Significant difference (*P* < 0.05) between number of total variant calls for the top 10% and those for the bottom 10% of each parameter is indicated by asterisk (*).

Our aim in the current study was to investigate whether sequencing results are affected by quality differences during the library preparation. We thus tested if QC during the diverse library preparation protocol can foresee any impact on the quality of the resulting sequencing library. To do so, we first examined the statistical distributions of all assessed QC parameters over a set of 581 patient samples undergoing SureSelect target enrichment (referred as “main cohort” hereafter). The QC steps examined include initial DNA-shearing and cleanup (QC1), pre-PCR and clean-up (QC2), as well as post-PCR and clean-up (QC3). Figure 1 depicts Violin plots of the distributions of the following parameters at each QC step: DNA concentration, largest fragment size, and smallest fragment size, which are all approximately normal.

**Figure 2:**
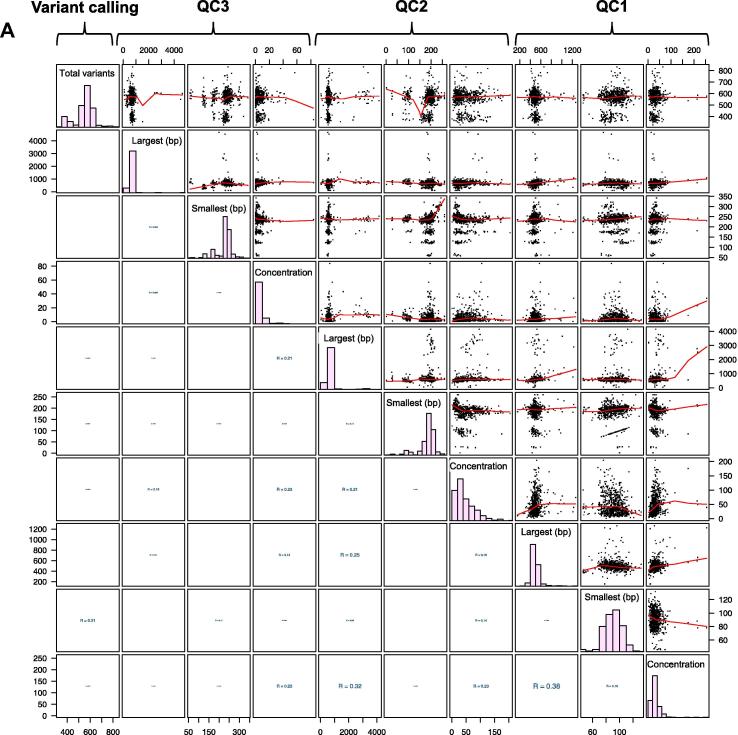

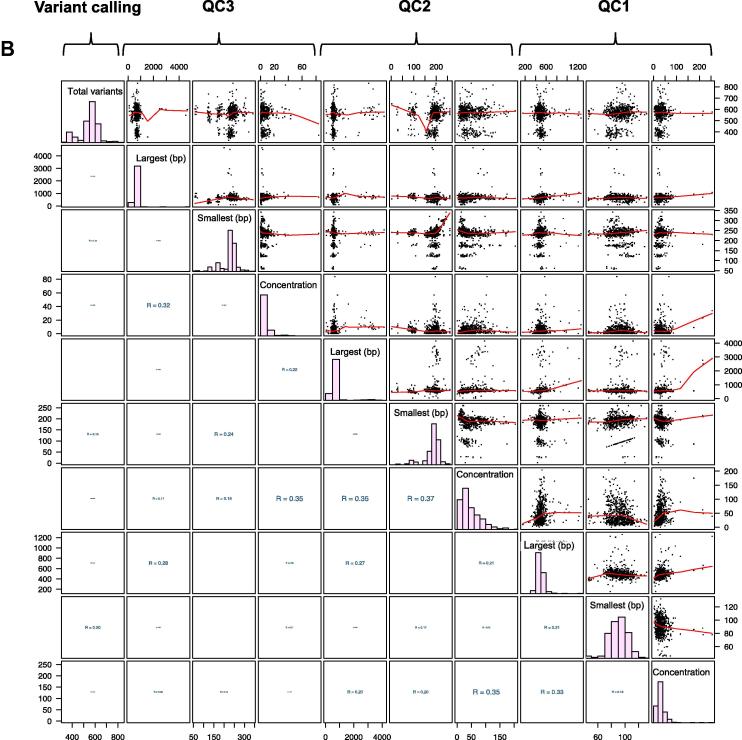
**QC-correlation analysis** The diagonal of the 10 × 10 matrix shows the histogram of total variants for the independent measurements of concentration, largest fragment length, and smallest fragment length at 3 QC steps (QC1, QC2, and QC 3). The lower triangular part of the matrix displays the absolute value of the Pearson correlation coefficients between the pair of parameters (**A**). The lower triangular part displays the absolute value of the Spearman correlation coefficients and the upper triangular part displays the Kendall correlation coefficients (**B**). Strong correlations are indicated with larger font sizes, while smaller fonts are used for weak correlations. The upper triangular part of the matrix displays the scatter plots between the pair of parameters, together with a best-fit line (red). The parameter shown on the *x*-axis of the scatter plot is given below inside the diagonal, and that on the *y*-axis is given leftward inside the diagonal. Correlation coefficient ranges 0–1 (no linear correlation: 0–0.5; weak correlation: 0.5–0.8; strong correlation: 0.8–1).

Then we tested by Pearson correlation, as well as Spearman and Kendall, whether the aforementioned parameters measured at different steps of the library preparation protocol exert significant impact on the quality of the resulting sequencing library. Surprisingly, we did not find any obvious correlation between the different QC steps and library QC measures, all correlation coefficients were below 0.4 (Figure 2). Next, we calculated linear correlations of the different QC steps with the total number of detected sequence variants as an indicator of final sequence quality. The lower triangular part of the matrix in Figure 2A shows the absolute values of the Pearson correlation coefficients between every possible pair of parameters, whereas the upper triangular part shows the scatter plots. Figure 2B shows the absolute values of the Spearman correlation coefficients below the diagonal and the absolute values of the Kendall correlation coefficients above the diagonal. Again, we did not detect obvious correlations.

### Robustness of library preparation for NGS

To further underline these findings, we applied Mann–Whitney *U*-test and Székely’s distance correlation on the total number of variant calls to rule out the possibility of undetected correlation in outliers and dependency of variant calls. As shown in Figure 1, the horizontal red bars indicate the total number of variant calls on the top 10% of the study population for each parameter and the blue bars on the bottom 10%, respectively. At QC1, the numbers of variant calls differ significantly between the bottom 10% population and top 10% population in terms of the smallest fragment size obtained (*P* = 0.04; *U*-test). According to Székely’s distance correlation coefficients (Table 1), there is a weak dependency between the smallest fragment size and number of variant calls at QC1 (*R* = 0.25), whereas the distance correlation coefficients are consistently less than 0.2 for the remaining parameters. This indicates that the number of total variant calls is reasonably independent from the parameters at all three QC steps. These data demonstrate that except the smallest fragment size at QC1, our indicator of sequence quality does not correlate well to the QC parameters. Even in the 10% populations at both extremes, no statistically significant influence of QC parameters on the final datasets has been observed.

**Figure 3:**
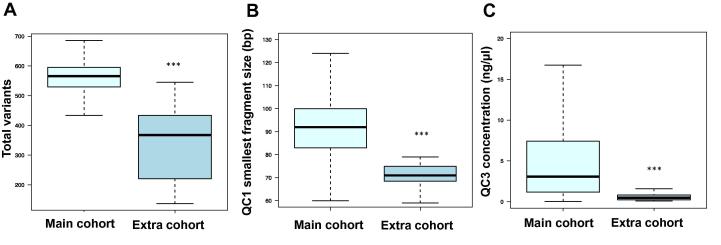
**Comparison of study cohorts** The boxplots illustrate the difference between the main cohort and extra cohort in terms of number of total variant calls (**A**), QC1 smallest fragment size (**B**), and QC3 concentration (**C**), respectively. Significant difference between main cohort and extra cohort is indicated with asterisks (*P* < 0.001, *t*-test).

**Figure 4:**
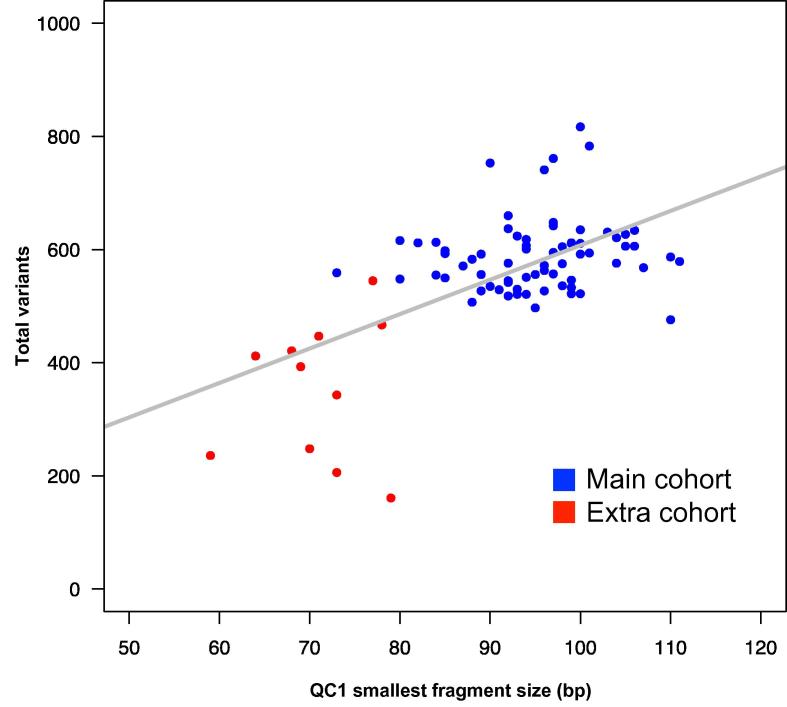
**Pearson correlation of QC1 fragment size and total variants** The scatter plot of QC1 smallest fragment size *vs.* number of total variant calls is shown. The red dots represent the extra cohort, while the blue dots represent main cohort samples who share the same geographic background as the extra cohort. The gray line is the best-fit line (Pearson correlation; *R* = 0.61; *P* < 0.001). The extra cohort has QC1 smallest fragment size less than 80 bp showing large deviation in number of total variants.

To further examine the dependency between number of variant calls and smallest fragment size at QC1, we analyzed 11 samples that were previously excluded from the sequencing study due to aberrant QC parameters (referred as “extra cohort” hereafter). Figure 3 exemplarily shows that the extra cohort differs significantly from our main cohort in the number of total variants, the smallest fragment size at QC1, and concentration at QC3 (*P* < 0.001; *t*-test). By combining the extra cohort with our main cohort, the coefficients of distance correlation fluctuate by up to |Δ*R*| = 0.03 (Table 1). In particular, the coefficient between QC1 smallest fragment size and total variants goes up from 0.25 to 0.28. *U*-test significance on the 10% extremes also becomes greater with *P* value reduced from 0.04 to 0.003, indicating that total variants depend on the smallest fragment size at QC1. To exclude a population effect on the number of total variants detected in the main and the extra cohorts, we selected from our European cohort only those samples having the same geographic background (*n* = 69) for correlation analysis. As a result, we observed a visible Pearson correlation (*R* = 0.61) between QC1 smallest fragment size and number of total variants (Figure 4). In particular, we observe that when QC1 smallest fragment size goes below 80 bp, the total variant count falls below its population stratum from the main cohort and fluctuates widely.

## Conclusion

The presented data indicate that the target enrichment protocol adopted in this study in combination with 2 × 100 bp sequencing by synthesis (HiSeq2000, Illumina) technology seems mostly affected by the initial QC and the smallest fragment size (<80 bp). Our data highlight the importance of a high quality input DNA and careful evaluation of the QC1 step (Figure 4). The fact that the 10% extreme tests at QC2 and QC3 steps are not affected by the extra cohort (Figure 1) suggests that the later stages of library preparation have good tolerances toward variations in quality. One might argue that sequence quality is more than the number of variants called, which was used as the surrogate of final data quality in this study. Although this is a potential limitation of the current study, the number of called variants still provides a robust overall assessment and is a good indicator of abnormalities in sequence data [7], [8], [9].

In summary, we postulate that a considerable variance in QC during target enrichment and library preparation is well tolerated within the boundaries indicated above (DNA after QC1 > 80 bp).

## Material and methods

### Data source

We used QC and sequencing data from a large-scale target enrichment sequencing study, lately performed by us [3]. Detailed information on the design of the 0.5 mb sized target region, hybridization-based target enrichment (SureSelect), 2 × 100 bp sequencing process (Illumina, HiSeq 2000), and data analysis can be found in the supplements of our previous report [3].

### Quality control

The quality of DNA library is tested at three major steps during the library preparation protocol. This is to ensure that the modifications of the DNA molecules introduced in the preceding step are successful and that enough material is left for the following modification steps. The first QC step ensures that the DNA is sheared at the right size and that enough DNA is left after a first cleanup with Ampure XP beads. The second QC step ensures that a successful adapter ligation is achieved and sufficient adapter-ligated DNA remained for the upcoming hybridization. The third QC step verifies that enough DNA is retained from the hybridization and amplification steps in a second PCR and that the size of the resulting final library is in the optimum range for paired-end sequencing.

Parameters that are quantified from readings of the Bioanalyzer (DNA 1000 assay) (Agilent; Waldbronn, Germany) platform include amount of sheared DNA and the smallest and largest fragment size of the sheared DNA; the amount of DNA after the pre-PCR and the smallest and largest fragment sizes of the resulting DNA; as well as the amount of final library and the smallest and largest fragment size of remaining DNA. QC was performed after the initial DNA-shearing and cleanup (QC1), after the pre-PCR and clean-up (QC2), and from the final library after post-PCR and clean-up (QC3).

### Statistical analysis

To examine the correlation between parameters, Pearson, Spearman, and Kendall correlation coefficients were computed for each pair of parameters. *U*-test [10] was performed on the number of variant calls for the top 10% and bottom 10% outliers for each parameter. Distance correlation was computed to determine independencies between the parameters [11] using ‘dcor’ command in package ‘energy’ [12]. Distance correlation coefficient is zero if and only if the variables are completely independent. *t*-test was performed between the main cohort and extra cohort. All computations are carried out in R (version 3.2). For distance correlation, ‘dcor’ command in package ‘energy’ was used [12].

## Authors’ contributions

RN, JH performed the quality control, library preparation, and sequencing. AL, DO, SM, FSH, EK, and AK extracted and analyzed data. RN, JH, and BM planned the study. RN, JH, AL, and BM wrote the manuscript. All authors read and approved the final manuscript.

## Competing interests

The authors have declared that there are no competing interests.

## Figures and Tables

**Table 1:** Szekely’s distance correlation coefficients in relation to total variants at different QC steps

**Parameters**		**Distance correlation coefficients**	***P* value**	**Δ coefficient with extra cohort**
QC1	Largest fragment size (bp)	0.11	<0.05	0.03
Concentration (ng/μl)	0.10	<0.05	−0.01
Smallest fragment size (bp)	0.25	<0.01	0.03
QC2	Largest fragment size (bp)	0.07	=0.325	0
Concentration (ng/μl)	0.15	<0.05	0
Smallest fragment size (bp)	0.19	<0.01	0.03
QC3	Largest fragment size (bp)	0.15	<0.01	0.02
Concentration (ng/μl)	0.15	<0.01	−0.03
Smallest fragment size (bp)	0.15	<0.01	0.01

*Note:* Distance correlation coefficient is zero if and only if the variable pair is independent.
